# Survival outcomes and mobilization during venovenous extracorporeal membrane oxygenation: a retrospective cohort study

**DOI:** 10.3389/fmed.2023.1271540

**Published:** 2023-09-28

**Authors:** Felix A. Rottmann, Christian Noe, Xavier Bemtgen, Sven Maier, Alexander Supady, Tobias Wengenmayer, Dawid L. Staudacher

**Affiliations:** ^1^Department of Medicine IV – Nephrology and Primary Care, Faculty of Medicine and Medical Center, University of Freiburg, Freiburg, Germany; ^2^Interdisciplinary Medical Intensive Care, Faculty of Medicine and Medical Center, University of Freiburg, Freiburg, Germany; ^3^Department of Cardiology and Angiology I, Faculty of Medicine and Medical Center, University of Freiburg, Freiburg, Germany; ^4^Department of Cardiovascular Surgery, Faculty of Medicine and Medical Center, University of Freiburg, Freiburg, Germany

**Keywords:** extracorporeal membrane oxygenation, muscle weakness, intensive care unit-acquired weakness, mobilization, acute respiratory distress syndrome, intensive & critical care, early ambulation, rehabilitation

## Abstract

**Introduction:**

Venovenous extracorporeal membrane oxygenation (V-V ECMO) can be considered in critically ill patient in severe pulmonary failure. However, the mobilization of patients on V-V ECMO can be challenging due to logistic and safety concerns. This study aimed to investigate whether 30 days survival was improved in patients who were mobilized during V-V ECMO support.

**Methods:**

We conducted a retrospective cohort all-comer study that included all patients cannulated for V-V ECMO at a single center. Patients with a V-V ECMO duration below 24 h were excluded from the analysis. The patients were grouped based on the ICU mobility scale documented during V-V ECMO support. The primary endpoint was 30 days survival, and secondary endpoints included weaning from ECMO and mechanical ventilation, as well as hospital survival.

**Results:**

A total of 343 patients were included in the study, with a median age of 56 years and 32% were female. Among them, 28% had chronic lung disease. The ICU mobilization scale ≥2 during ECMO was documented in 62/343 (18%) patients. There were no significant differences in age, gender and preexisting lung disease. Duration of ICU stay (13.1 vs. 15.6 days), time on ECMO (186 vs. 190 h) and mechanical ventilation (11.2 vs. 13.6 days) were slightly shorter in patients with ICU mobility scale <2 compared to those with ≥2 (all *p* = 0.0001). However, patients with ICU mobilization scale ≥2 showed significantly better 30 days survival (71.0 vs. 48.0%, OR 2.6 (1.5 to 4.8), *p* = 0.0012) compared to those with <2. In the ≥2 mobility scale group, a significantly higher number of patients were successfully weaned from the ventilator (61.3 vs. 46.6%, OR 1.8 (1.0 to 3.2), *p* = 0.049). A stronger correlation was observed between more intense mobilizations, such as being in a standing position (OR 5.0 (1.7 to 14.0), *p* = 0.0038), and higher 30 days survival.

**Conclusion:**

The findings of this study suggest that active mobilization during V-V ECMO support is associated with improved 30 days survival and successful weaning from the respirator. Incorporating mobilization as part of the therapeutic approach during ECMO support may offer potential benefits for critically ill patients.

## Introduction

Patients cannulated for venovenous extracorporeal membrane oxygenation (V-V ECMO) support are often in critical condition and experience extended hospitalizations on intensive care units (ICU) (1). They also require prolonged periods of invasive mechanical ventilation (2), and face high rates of hospital mortality (3).

Poor outcome of patients on V-V ECMO is multi-factorial and partly determined by the severity of the underlying pulmonary failure indicating ECMO (4) and complications secondary to the extracorporeal support (5). In addition, hospitalization on ICU itself comes with the risk of development of an ICU-acquired weakness (6). In heathy individuals, 6 weeks of bed rest results in 30% loss of muscle strength due to inactivity (7). In context of sedation, relaxation, or katabolic conditions typical to severe pulmonary failure, the muscle loss is much more pronounced (8). In critically ill patients, the incidence of ICU-acquired weakness is reported roughly in 40% (9) and is associated with adverse outcome including a prolonged hospitalization, impaired weaning and increased risk of death (10). Reports on ICU-acquired weakness in V-V ECMO support suggest even higher rates of up to 80% of patients (11). It is accepted that an ICU-acquired weakness also correlates with long-term outcome (12, 13).

The ABCDEF bundle in critical care treatment was designed to improve patient recovery, combats the issues discussed above and includes spontaneous awakening and breathing trials as well as early mobilization and exercise for all ICU patients (14): mobilization on ICU however can be challenging and requires an experienced team (15). When executed correctly, mobilization might improve outcome by reducing the incidence of ICU-acquired weakness (16, 17).

In context of V-V ECMO evidence is much more limited (18). Few trials investigated mobilization on ECMO, data available however suggests mobilization is feasible (19–21). Data from the ELSO registry even suggests a significantly lower probability of death (16) in mobilized patients. Randomized data from the notable ANZICS trial in mechanically ventilated patients however showed similar mortality with a signal of harm in mobilized patients (22).

The role of mobilization in V-V ECMO therefore remains a topic of debate. To address this, we sought to investigate the null hypothesis that patients without mobilization during V-V ECMO support and those with mobilization experience similar 30 days survival rates. The study aimed to test this hypothesis using our dataset, with the primary endpoint being 30 days survival, and secondary endpoints being weaning from V-V ECMO and invasive mechanical ventilation (IMV) as well as hospital survival.

## Methods

### Registry

This is a retrospective cohort study. Data presented follows the STROBE guideline (23) for retrospective registries. We investigate an all-comer collective of all patients cannulated for V-V ECMO at the Interdisciplinary Medical Intensive Care (IMIT) unit of the university hospital Freiburg, Germany. Patients were detected by computerized search for the OPS (German operation and procedure classification system) code for ECMO (8-852). Type of support (venovenous, venoarterial or mixed) was reviewed on a case-by-case basis. Inclusion criteria for this registry were age at least 18 years at cannulation, primary venovenous support (excluding veno-venoarterial and veno-arterial ECMO), and a duration of V-V ECMO support of at least 24 h. The ethics committee of the University of Freiburg (file number 21-1683) approved this registry.

### V-V ECMO center standards

At our center, patient selection for V-V ECMO support is based on established criteria (24, 25). Information on our local ECMO policy can be found here (26, 27). Specifically to this manuscript, we encourage mobilization of ECMO all patients. To ensure patient safety during transfers, a set of standardized operating procedures have been implemented, and interdisciplinary teams are assembled and trained accordingly. For patients experiencing acute pulmonary failure, the early adoption of prone positioning is strongly encouraged whenever it is feasible (26, 28). If not conflicting with prone positioning, we encourage mobilizing patients out of their beds and into chairs, even if they are under mechanical ventilation or ECMO support. This approach is aimed at mitigating the risk of ICU-acquired weakness and optimizing respiratory function (29). Decisions regarding the most appropriate mobilization technique are made on a case-by-case basis at the bedside, considering the individualized needs and conditions of each patient.

### Data acquisition and group allocation

The data for this study derives from information from electronic patient files and discharge letters collected manually without a computerized data acquisition. The duration of Extracorporeal Membrane Oxygenation was measured from the time of ECMO implantation until either successful weaning of ECMO support or the patient’s death. For mobilization, only episodes on ECMO support were registered. Best mobilization events during ECMO support were paramount for group allocation, e.g., one mobilization to a standing position qualified a patient to be allocated to this group even if most mobilizations were only to a chair.

Patients were grouped for statistical analysis using two complementing systems:

1. ICU mobility scale [as proposed in literature on mobilization (16, 20, 30, 31)]
  - Patients with mostly passive mobilization in bed (ICU mobility scale 0 or 1) were compared to those with more active mobilization (ICU mobility scale 2 or more) (32) (Table 1).
2. Best mobilization event on ECMO support
  - Groups were created following the ICU mobility scale levels 0–4 corresponding to mobilization levels: no mobilization other than prone positions (Group: None), mobilization in bed (In bed), sitting on edge of bed (Edge of bed), mobilization to a chair (Chair) or to a standing position (Standing) (Table 2).

**Table 1:** Patients characteristics and endpoints by ICU mobility scale under V-V ECMO.

Baseline characteristics	Total (*n* = 343)	ICU mobility scale <2 (*n* = 281)	ICU mobility scale ≥2 (*n* = 62)	*p*-value
Percentage of patients	100	82	18	
Age	55 (45–64)	55 (45–64)	57 (42–63)	0.9014^a^
Female gender	108 (31.5%)	90 (32%)	18 (29%)	0.7628^b^
BMI	25.6 (23.7–30.5)	24.8 (23.5–30.2)	26.8 (23.8–30.6)	0.2358^a^
Preexisting conditions				
Hypercholesterolemia	41 (12%)	34 (12.1%)	7 (11.3%)	0.9999^b^
Nicotine use disorder	109 (31.8%)	89 (31.7%)	20 (32.3%)	0.9999^b^
Coronary heart disease	37 (10.8%)	29 (10.3%)	8 (12.9%)	0.5058^b^
Hypertension	124 (36.2%)	98 (34.9%)	26 (41.9%)	0.3092^b^
Liver cirrhosis or chronic hepatitis	21 (6.1%)	20 (7.1%)	1 (1.6%)	0.1425^b^
Chronic kidney disease	23 (6.7%)	20 (7.1%)	3 (4.8%)	0.7788^b^
Diabetes mellitus	49 (14.3%)	40 (14.2%)	9 (14.5%)	0.9999^b^
Oncological disorders	59 (17.2%)	52 (18.5%)	7 (11.3%)	0.1972^b^
Immunodeficiency	93 (27.1%)	83 (29.5%)	10 (16.1%)	**0.0394**^**b**^
Chronic lung disease	97 (28.3%)	79 (28.1%)	18 (29%)	0.8772^b^
CPR within 48 h before EMCO	32 (9.3%)	30 (10.7%)	2 (3.2%)	**0.0890**^**b**^
Respiratory situation before ECMO				
Horowitz index	70 (58–93)	71 (59–96)	64 (51–79)	**0.0038**^**a**^
pO2 – arterial (mmHg)	65 (58–75)	66 (58–76)	62 (47–73)	**0.0173**^**a**^
FiO2	1.0 (0.8–1.0)	1 (0.8–1)	1 (0.9–1)	0.1662^a^
pCO2 – arterial (mmHg)	56 (46–73)	57 (46–73)	50 (43–72)	0.2392^a^
pH – arterial	7.3 (7.2–7.3)	7.2 (7.2–7.3)	7.3 (7.2–7.4)	**0.0002**^**a**^
Peak inspiratory pressure ≥ 42 cm H_2_O	33 (9.6%)	30 (10.7%)	3 (4.8%)	0.2325^b^
ICU stay				
Duration of ICU stay from ECMO d1 [d]	13.7 (8.7–26.8)	12.9 (7.3–21.5)	25.7 (13.8–53.4)	**0.0001**^**a**^
ECMO runtime [h]	190 (114–361)	173 (100–288)	577 (243–1009)	**0.0001**^**a**^
Mechanical ventilation [d]	11.8 (6.7–23.8)	10.9 (6.4–19.5)	25.4 (10.1–50.0)	**0.0001**^**a**^
Endpoint				
30-days survival	179 (52.2%)	135 (48.0%)	44 (71.0%)	**0.0012**^**b**^
ICU survival	160 (46.6%)	127 (45.2%)	33 (53.2%)	0.2636^b^
Hospital survival	159 (46.4%)	126 (44.8%)	33 (53.2%)	0.2612^b^
ECMO weaning	197 (57.4%)	159 (56.6%)	38 (61.3%)	0.5709^b^
Vent. Weaning	169 (49.3%)	131 (46.6%)	38 (61.3%)	0.0489^b^

Data given in median (interquartile range) or in number of patients (percentage of group). *p*-values are calculated between groups using either ^a^Mann–Whitney U-test or ^b^Fishers Exact test.BMI, body-mass-index; CPR, cardiopulmonary resuscitation; ECMO, extracorporeal membrane oxygenation; Vent., ventilation. Bold values indicate a *p*-value < 0.05.

**Table 2:** Patients characteristics and endpoints by best mobilization under V-V ECMO.

Baseline characteristics	Total (*n* = 343)	None (*n* = 179)	In bed (*n* = 102)	Edge of bed (*n* = 9)	Chair (*n* = 33)	Standing (*n* = 20)	*p*-value
Percentage of patients	100	52	30	3	10	6	
Age	55 (45–64)	54 (44–61)	59 (46–67)	50 (33–66)	56 (50–64)	58 (36–63)	0.2072^c^
Female gender	108 (31.5%)	64 (35.8%)	26 (25.5%)	3 (33.3%)	11 (33.3%)	4 (20%)	0.3424^d^
BMI	25.1 (23.6–30.2)	24.5 (23.5–30.1)	26.1 (23.9–30.6)	28.4(24.8–39.6)	26.5(23.9–31.8)	26.0 (21.4–29.4)	0.1174^c^
Preexisting conditions							
Hypercholesterolemia	41 (12%)	20 (11.2%)	14 (13.7%)	2 (22.2%)	3 (9.1%)	2 (10%)	0.8018^d^
Nicotine use disorder	109 (31.8%)	63 (35.2%)	26 (25.5%)	3 (33.3%)	11 (33.3%)	6 (30%)	0.5746^d^
Coronary heart disease	37 (10.8%)	20 (11.2%)	9 (8.8%)	1 (11.1%)	5 (15.2%)	2 (10%)	0.8937^d^
Hypertension	124 (36.2%)	61 (34.1%)	37 (36.3%)	3 (33.3%)	16 (48.5%)	7 (35%)	0.6355^d^
Liver cirrhosis or chronic hepatitis	21 (6.1%)	18 (10.1%)	2 (2%)	0 (0%)	1 (3%)	0 (0%)	**0.0352**^**d**^
Chronic kidney disease	23 (6.7%)	11 (6.1%)	9 (8.8%)	1 (11.1%)	2 (6.1%)	0 (0%)	0.6339^d^
Diabetes mellitus	49 (14.3%)	27 (15.1%)	13 (12.7%)	0 (0%)	6 (18.2%)	3 (15%)	0.6975^d^
Oncological disorders	59 (17.2%)	34 (19%)	18 (17.6%)	0 (0%)	4 (12.1%)	3 (15%)	0.5655^d^
Immunodeficiency	93 (27.1%)	56 (31.3%)	27 (26.5%)	3 (33.3%)	4 (12.1%)	3 (15%)	0.1353^d^
Chronic lung disease	97 (28.3%)	50 (27.9%)	29 (28.4%)	3 (33.3%)	9 (27.3%)	6 (30%)	0.9966^d^
CPR within 48 h before EMCO	32 (9.3%)	23 (12.8%)	7 (6.9%)	0 (0%)	2 (6.1%)	0 (0%)	0.1494^d^
Respiratory situation before ECMO							
Horowitz index	70 (58–93)	72 (60–107)	69 (58–87)	70 (61–102)	63 (49–77)	65 (46–96)	**0.0091**^**c**^
pO2 - arterial [mmHg]	65 (58–75)	66 (58–78)	64 (56–71)	70 (59–80)	62 (44–71)	61 (45–73)	**0.0164**^**c**^
FiO2	1.0 (0.8–1.0)	1 (0.8–1)	1 (0.85–1)	1 (0.9–1)	1 (1–1)	1 (0.8–1)	0.4644^c^
pCO2 - arterial [mmHg]	56 (46–73)	56 (46–72)	59 (45–78)	51 (41–115)	54 (47–71)	48 (40–71)	0.6284^c^
pH - arterial	7.3 (7.2–7.3)	7.2 (7.2–7.3)	7.3 (7.2–7.3)	7.3 (7.2–7.4)	7.4 (7.2–7.5)	7.3 (7.3–7.4)	**0.0030**^**c**^
Peak inspiratory pressure ≥ 42 cm H2O	33 (9.6%)	24 (13.4%)	6 (5.9%)	1 (11.1%)	0 (0%)	2 (10%)	0.0869^d^
ICU stay							
Duration of ICU stay from ECMO d1 [d]	13.7 (8.7–26.8)	11.0 (5.9–18.0)	17.0 (9.5–29.7)	26.7 (14.4–57.3)	17.8(10.8–42.8)	40.8(21.7–72.6)	**0.0001**^**c**^
ECMO runtime [h]	7.9 (4.7–15.0)	147 (80–239	230 (147–413)	615 (169–852)	422 (220–778)	782 (407–1664)	**0.0001**^**c**^
Mechanical ventilation [d]	11.8 (6.7–23.8)	9.5 (5.3–15.3)	14.8 (8.7–26.4)	26.7 (10.0–38.9)	19.1 (10.5–45.2)	38.1 (5.7–72.6)	**0.0001**^**c**^
Endpoints							
30 days survival	179 (52.2%)	80 (44.7%)	55 (53.9%)	6 (66.7%)	22 (66.7%)	16 (80%)	**0.0077**^**d**^
ICU survival	160 (46.6%)	78 (43.6%)	49 (48.0%)	4 (44.4%)	17 (51.5%)	12 (60%)	0.6406^d^
Hospital survival	159 (46.4%)	78 (43.6%)	48 (47.1%)	4 (44.4%)	17 (51.5%)	12 (60%)	0.6553^d^
ECMO weaning	197 (57.4%)	103 (57.5%)	56 (54.9%)	5 (55.6%)	19 (57.6%)	14 (70%)	0.8136^d^
Vent. Weaning	169 (49.3%)	80 (44.7%)	51 (50.0%)	5 (55.6%)	19 (57.6%)	14 (70%)	0.1981^d^

Data given in median (interquartile range) or in number of patients (percentage of group). *p*-values are calculated between groups using either ^c^Kruskal–Wallis test or ^d^Chi-Square test. BMI, body-mass-index; CPR, cardiopulmonary resuscitation; ECMO, extracorporeal membrane oxygenation; Vent., ventilation. Bold values indicate a *p*-value < 0.05.

### Statistical methods

In this registry, data was manually collected and pseudonymized, and the pooled data was subjected to analysis. The groups were created based on the mobilization events during ECMO support. To perform statistical analysis on continuous data, we assessed whether the data followed a normal distribution using the D’Agostino & Pearson test (GraphPad Prism 10.0, GraphPad Software, San Diego, CA, United States). None of the data was normally distributed. For non-normally distributed data the Kruskal–Wallis test and the Mann–Whitney U test were employed (GraphPad Prism 10.0, GraphPad Software, San Diego, CA, United States). For categorical data, we used the Chi-squared test and Fisher’s exact test (GraphPad Prism 10.0, GraphPad Software, San Diego, CA, United States) as statistical methods.

The Kaplan–Meier survival curve was constructed to compare patients by best mobilization at any point during the extracorporeal membrane oxygenation treatment. The analysis included surviving patients who were followed up for less than 30 days because of discharge and their data were considered as representing a 30 days follow-up period for the purposes of creating the graph.

## Results

### Study population

A total of 343 patients were treated with V-V ECMO for ≥24 h between October 2010 and May 2021 (cannulation window). An ICU mobility scale of ≥2 was reached in 62 patients while 281 stayed <2.

In total 164 patients received some form of mobilization while 179 did not. Patients were defined by best mobilization under ECMO. The most frequently reached stage of mobilization was in bed mobilization (*n* = 102). 53 patients were mobilized out of bed to a chair (*n* = 33) or to a standing position (*n* = 20). Out of 179 patients without mobilization 59 received prone positioning. All groups were similar regarding demographics, preexisting conditions and respiratory situation before ECMO cannulation (Tables 1, 2).

ICU stays lasted for median 13.3 days (IQR 7.2–25.9) with mechanical ventilation for median 11.6 days (IQR 6.2–23.6) and an ECMO runtime of median 187 h (102–356). Duration of ICU stay (13.1 vs. 15.6 days), time on ECMO (186 vs. 190 h) and mechanical ventilation (11.2 vs. 13.6 days) were slightly shorter in patients with ICU mobility scale <2 compared to those with ≥2 (all *p* = 0.0001). 137 ECMO runs lasted for >240 h, the longest being 2,329 h.

### Survival

**Figure 1:**
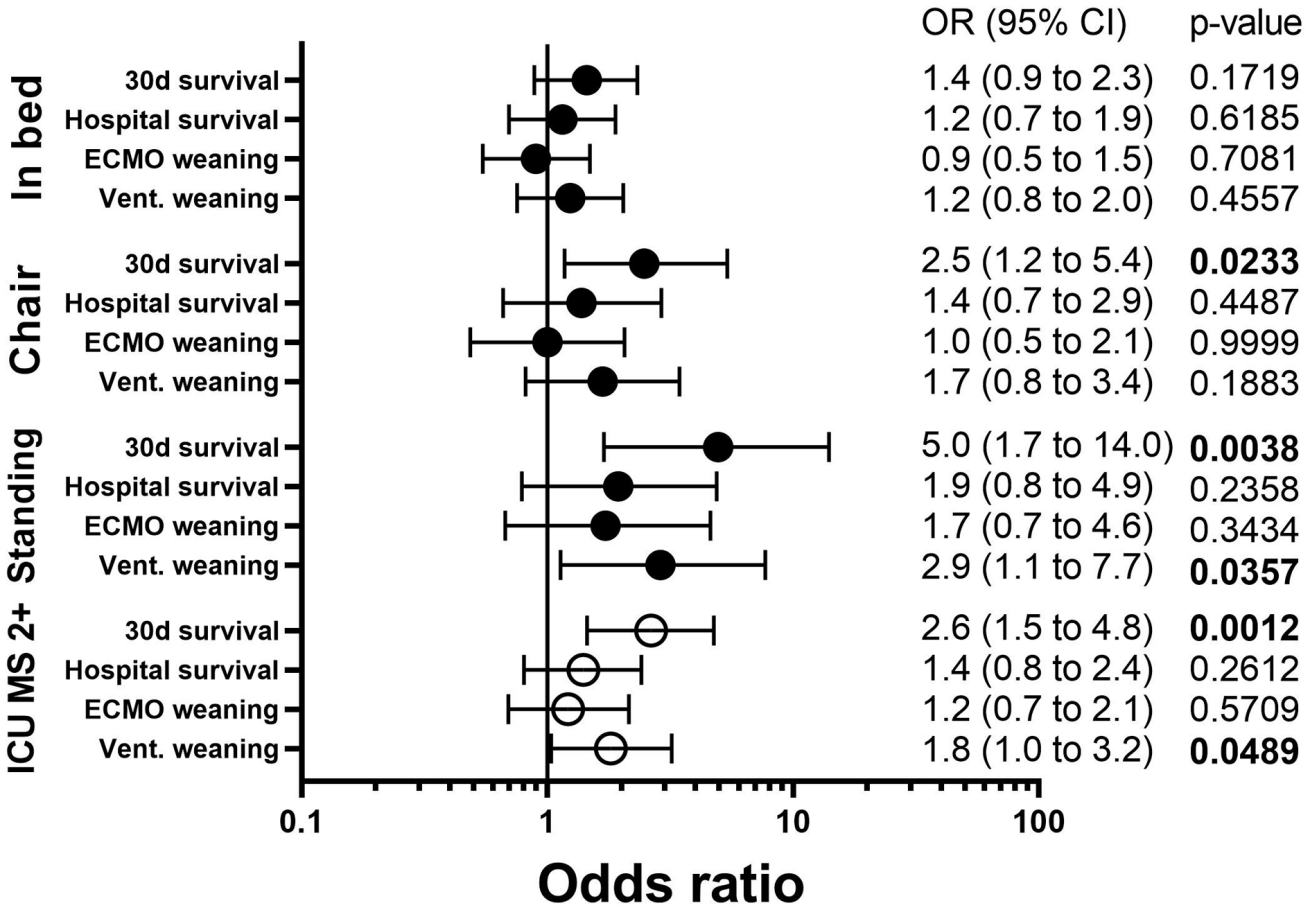
Outcome in V-V ECMO according to mobilization. Each mobilization (In bed, Chair, Standing) was compared to patients without mobilization (None) or to those with a lower ICU mobility scale (ICU MS 2+) and an OR for each outcome was calculated. A Odds ration >1 indicates better outcome with mobilization. OR, odds ratio; CI, confidence interval; ICU, intensive care unit; ICU MS 2+, ICU mobility scale ≥2; Vent., Ventilation.

30 days survival was significantly higher in patients with ICU mobility scale ≥2 (i.e., at least passive mobilization into a chair) to <2 (71.0 vs. 48.0%, OR 2.6, (1.5 to 4.8), *p* = 0.0012). Ventilation weaning was also significantly higher in ICU mobility scale ≥2 (61.3 vs. 46.6%, OR 1.8 (1.0 to 3.2), *p* = 0.0489). Hospital survival was not influenced significantly by ICU mobility scale ≥2 (53.2 vs. 44.8%, OR 1.4 (0.8 to 2.4), *p* = 0.2612) as ECMO weaning was not either (61.3 vs. 56.6%, OR 1.2 (0.7 to 2.1), *p* = 0.5709) (Table 1 and Figure 1).

More detailed analysis of the different levels of the ICU mobility scale confirmed that 30 days survival was higher in patients mobilized into a chair (66.7 vs. 52.2%, OR 2.5 (1.2 to 5.4), *p* = 0.0233) or to a standing position (80.0 vs. 52.2%, OR 5.0 (1.7 to 14.0), *p* = 0.0038) while in bed mobilization was not sufficient to improve survival (53.9 vs. 52.2%, OR 1.4 (0.9 to 2.3), *p* = 0.1719). Hospital survival was not influenced significantly by in bed mobilization (*p* = 0.6185), mobilization to a chair (*p* = 0.4487) or standing position (*p* = 0.2358) but all showed odds ratios pointing towards better survival (OR 1.2 vs. 1.4. vs. 1.9, see Table 2 and Figure 1). For a full analysis of all levels of the ICU mobility scale see Supplementary Figure S1.

**Figure 2:**
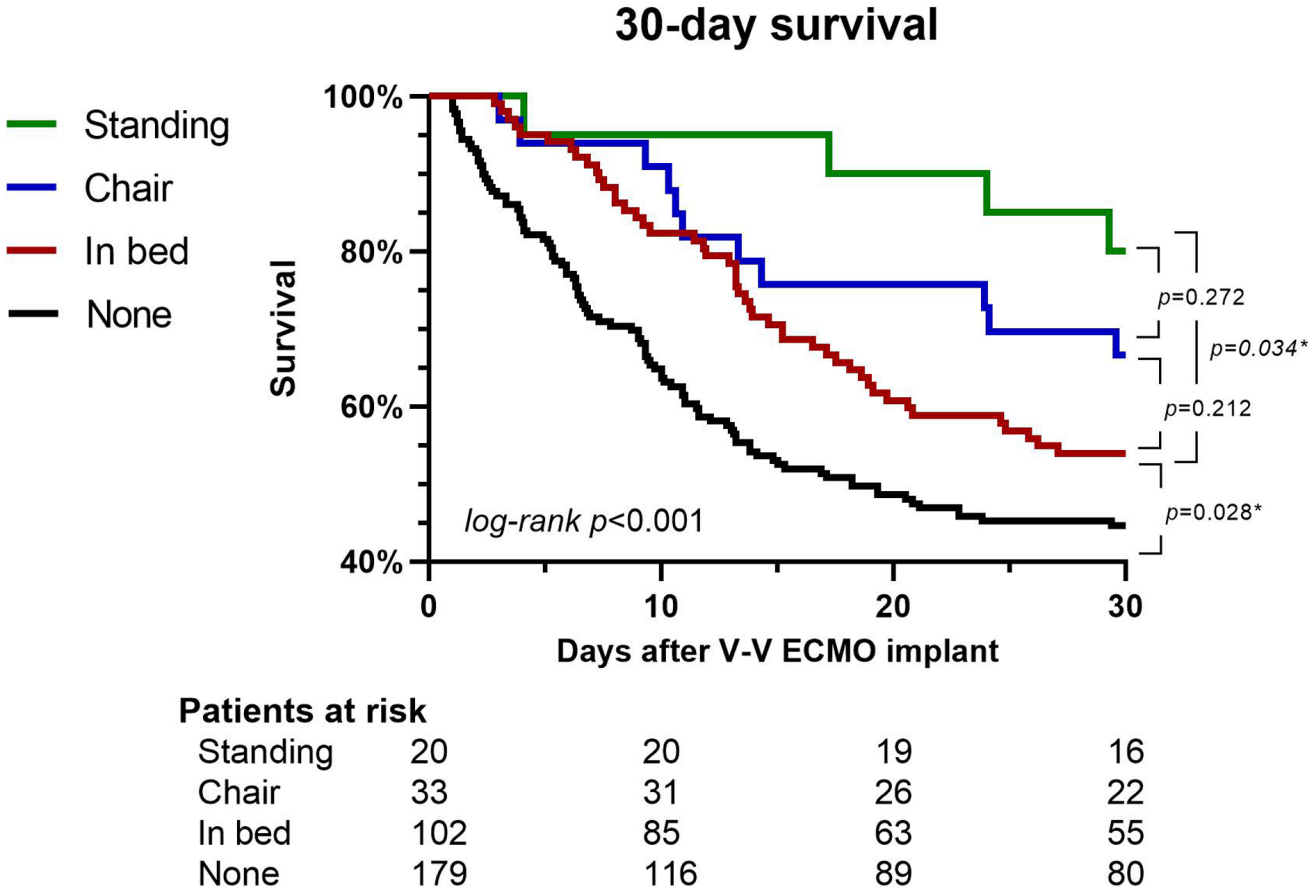
30 days survival according to mobilization. Patients not mobilzed (black), receiving in bed mobilization (red), out of bed mobilization to a chair (blue) and those mobilized to a standing position (green) are shown. More intensive mobilzation corresponded to higher 30 days survival. V-V ECMO, veno-venous extracorporeal membrane oxygenation.

Kaplan–Meier 30 days survival analysis showed higher survival rates in patients receiving in bed mobilization compared to no mobilization (*p* = 0.028). While differences between in bed mobilization and mobilization to chair (*p* = 0.212) and mobilization to chair and a standing position (*p* = 0.272) were not significant, mobilization to a standing position resulted in significantly higher survival than in bed mobilization (*p* = 0.034, see Figure 2).

An analysis comparing all patients receiving in bed mobilization to all who did not (i.e., patients with less mobilization or those that skipped in bed mobilization on their course to higher levels of mobilization) confirmed this trend as in bed mobilized patients showed significantly higher 30 days survival than the others (60.9 vs. 45.3%, OR 1.8 (1.2 to 2.9), *p* = 0.0047, Supplementary Figure S2).

## Discussion

Mobilization during V-V ECMO improved outcome in this retrospective cohort study. More physically demanding mobilizations like a free stand improved the outcome even more.

This observations matches data from the ELSO registry suggesting better survival in patients mobilized on ECMO (16) and other smaller studies (33–35). Our data showed a significant improvement only on 30 days survival and only in those patients mobilized into the chair (active or passive) or in the stand. This might suggest that a higher intensity of training is important to improve outcome. A meta-analysis of literature on mobilization on ECMO (36) suggested that more intense mobilization increases outcome. This observation can be strengthened by our data and fits to data suggesting that especially out of bed mobilization improves outcome (37). Of course patients with a higher chance of survival also could have been more able to be mobilized rather than mobilization improving survival. The important aspect of causation cannot be addressed in a retrospective analysis.

When investigating hospital (and ICU) survival, no significant impact of mobilization on outcome was detected. This might be explained by the fact that for those patients with an ICU duration longer than 30 days, prognosis is predominantly determined by complications and not by the mobilizations during ECMO.

Data from the US suggests that health care costs in ECMO patients can also be reduced by physical activity (38). Nonetheless many patients suffer from barriers to mobilization such as unstable fractures (e.g., spinal), wounds after surgery, drainage systems or simply insufficient staff when mobilizing for example patients with a very high BMI.

Data on mobilization on V-V ECMO suggests that 22 to 35% of V-V ECMO patients predominantly at high volume ECMO centers are mobilized (16, 21). In our registry, 47.8% of patients receive some form of mobilization. Active mobilization on ECMO out of bed (which corresponds to an ICU mobility scale) of at least 2 (32), was documented in 18% of patients, only. Mobilization out of bed therefore was a rare event at our institution. Data suggests that barriers exist for mobilizing patients on ECMO primary connected to safety concerns (21, 30). Some registries suggest that mode of cannulation might facilitate mobilization (16, 27). Data on complications on ECMO however is promising (16, 33, 39). Also the fact that high volume centers mobilize more (16, 21, 33, 37) suggests that experience and training can counteract safety issues. In fact, there are data showing that mobilization was especially performed in sicker patients on ECMO and those with higher BMI (40).

A potential bias conflicting this research is the concern that patients who died early had no possibility to be mobilized. This is a common problem in retrospective data on mobilization found in literature, reporting longer ECMO duration in mobilized patients (33, 34, 38) or duration of mechanical ventilation (34, 35) and ICU stays (35). In our dataset, we report similar durations of ECMO, ICU stay and mechanical ventilation in mobilized and non-mobilized patients not hinting towards an observation bias.

## Conclusion

The findings of this study suggest that active mobilization during V-V ECMO support is associated with improved 30 days survival and successful weaning from the respirator. Incorporating mobilization as part of the therapeutic approach during ECMO support may offer potential benefits for critically ill patients.

## Data availability statement

The original contributions presented in the study are included in the article/Supplementary material, further inquiries can be directed to the corresponding author.

## Ethics statement

The studies involving humans were approved by Ethics committee of the University of Freiburg, Germany (file number 21-1683). The studies were conducted in accordance with the local legislation and institutional requirements. Written informed consent for participation was not required from the participants or the participants’ legal guardians/next of kin in accordance with the national legislation and institutional requirements.

## Author contributions

FR: Conceptualization, Data curation, Formal analysis, Funding acquisition, Visualization, Writing – original draft, Writing – review & editing. CN: Data curation, Formal analysis, Investigation, Writing – review & editing. XB: Formal analysis, Methodology, Writing – review & editing. SM: Formal analysis, Methodology, Writing – review & editing. AS: Formal analysis, Methodology, Writing – review & editing. TW: Formal analysis, Methodology, Writing – review & editing. DS: Conceptualization, Data curation, Formal analysis, Funding acquisition, Methodology, Project administration, Supervision, Validation, Visualization, Writing – original draft, Writing – review & editing.
