# Corrigendum: Survival outcomes and mobilization during venovenous extracorporeal membrane oxygenation: a retrospective cohort study

**DOI:** 10.3389/fmed.2024.1421367

**Published:** 2024-05-29

**Authors:** Felix A. Rottmann, Christian Noe, Xavier Bemtgen, Sven Maier, Alexander Supady, Tobias Wengenmayer, Dawid L. Staudacher

**Affiliations:** ^1^Department of Medicine IV–Nephrology and Primary Care, Faculty of Medicine and Medical Center, University of Freiburg, Freiburg, Germany; ^2^Interdisciplinary Medical Intensive Care, Faculty of Medicine and Medical Center, University of Freiburg, Freiburg, Germany; ^3^Department of Cardiology and Angiology I, Faculty of Medicine and Medical Center, University of Freiburg, Freiburg, Germany; ^4^Department of Cardiovascular Surgery, Faculty of Medicine and Medical Center, University of Freiburg, Freiburg, Germany

**Keywords:** extracorporeal membrane oxygenation, muscle weakness, intensive care unit-acquired weakness, mobilization, acute respiratory distress syndrome, intensive & critical care, early ambulation, rehabilitation

In the published article, there was an error in Tables 1, 2 as published. This error pertains to the continuous data presented in the primary manuscript. The median values and interquartile ranges were incorrect. It is crucial to note that the primary endpoint (mortality), figures, and all but one provided *p*-values (Table 1—Hypertension: *p* = 0.4740 was previously displayed and is incorrect. The correct *p*-value is 0.3092) remain unaffected, as they either do not involve continuous data or were calculated independently. The corrected Tables 1, 2 and its caption appear below.

**Table 1:** Patients characteristics and endpoints by ICU mobility scale under V-V ECMO.

**Baseline characteristics**	**Total (*n* = 343)**	**ICU mobility scale < 2 (*n* = 281)**	**ICU mobility scale ≥2 (*n* = 62)**	***p*-value**
Percentage of patients	100	82	18	
Age	55 (45–64)	55 (45–64)	57 (42–63)	0.9014^a^
Female gender	108 (31.5%)	90 (32%)	18 (29%)	0.7628^b^
BMI	25.1 (23.6–30.2)	24.8 (23.5–30.2)	26.8 (23.8–30.6)	0.2358^a^
**Preexisting conditions**				
Hypercholesterolemia	41 (12%)	34 (12.1%)	7 (11.3%)	0.9999^b^
Nicotine use disorder	109 (31.8%)	89 (31.7%)	20 (32.3%)	0.9999^b^
Coronary heart disease	37 (10.8%)	29 (10.3%)	8 (12.9%)	0.5058^b^
Hypertension	124 (36.2%)	98 (34.9%)	26 (41.9%)	0.3092^b^
Liver cirrhosis or chronic hepatitis	21 (6.1%)	20 (7.1%)	1 (1.6%)	0.1425^b^
Chronic kidney disease	23 (6.7%)	20 (7.1%)	3 (4.8%)	0.7788^b^
Diabetes mellitus	49 (14.3%)	40 (14.2%)	9 (14.5%)	0.9999^b^
Oncological disorders	59 (17.2%)	52 (18.5%)	7 (11.3%)	0.1972^b^
Immunodeficiency	93 (27.1%)	83 (29.5%)	10 (16.1%)	**0.0394** ^**b**^
Chronic lung disease	97 (28.3%)	79 (28.1%)	18 (29%)	0.8772^b^
CPR within 48 h before EMCO	32 (9.3%)	30 (10.7%)	2 (3.2%)	**0.0890** ^**b**^
**Respiratory situation before ECMO**				
Horowitz index	70 (58–93)	71 (59–96)	64 (51–79)	**0.0038** ^**a**^
pO_2_-arterial (mmHg)	65 (58–75)	66 (58–76)	62 (47–73)	**0.0173** ^**a**^
FiO_2_	1.0 (0.8–1.0)	1 (0.8–1)	1 (0.9–1)	0.1662^a^
pCO_2_-arterial (mmHg)	56 (46–73)	57 (46–73)	50 (43–72)	0.2392^a^
pH–arterial	7.3 (7.2–7.3)	7.2 (7.2–7.3)	7.3 (7.2–7.4)	**0.0002** ^**a**^
Peak inspiratory pressure ≥42 cm H_2_O	33 (9.6%)	30 (10.7%)	3 (4.8%)	0.2325^b^
**ICU stay**				
Duration of ICU stay from ECMO d1 (d)	13.7 (8.7–26.8)	12.9 (7.3–21.5)	25.7 (13.8–53.4)	**0.0001** ^**a**^
ECMO runtime (h)	190 (114–361)	173 (100–288)	577 (243–1009)	**0.0001** ^**a**^
Mechanical ventilation (d)	11.8 (6.7–23.8)	10.9 (6.4–19.5)	25.4 (10.1–50.0)	**0.0001** ^**a**^
**Endpoint**				
30-day survival	179 (52.2%)	135 (48.0%)	44 (71.0%)	**0.0012** ^**b**^
ICU survival	160 (46.6%)	127 (45.2%)	33 (53.2%)	0.2636^b^
Hospital survival	159 (46.4%)	126 (44.8%)	33 (53.2%)	0.2612^b^
ECMO weaning	197 (57.4%)	159 (56.6%)	38 (61.3%)	0.5709^b^
Vent. Weaning	169 (49.3%)	131 (46.6%)	38 (61.3%)	**0.0489** ^**b**^

Data given in median (interquartile range) or in number of patients (percentage of group). p-values are calculated between groups using either ^*a*^Mann–Whitney U-test or ^*b*^Fishers Exact test.

BMI, body-mass-index; CPR, cardiopulmonary resuscitation; ECMO, extracorporeal membrane oxygenation; Vent., ventilation. Bold values indicate a p-value < 0.05.

**Table 2:** Patients characteristics and endpoints by best mobilization under V-V ECMO.

**Baseline characteristics**	**Total (*n* = 343)**	**None (*n* = 179)**	**In bed (*n* = 102)**	**Edge of bed (*n* = 9)**	**Chair (*n* = 33)**	**Standing (*n* = 20)**	***p*-value**
Percentage of patients	100	52	30	3	10	6	
Age	55 (45–64)	54 (44–61)	59 (46–67)	50 (33–66)	56 (50–64)	58 (36–63)	0.2072^c^
Female gender	108 (31.5%)	64 (35.8%)	26 (25.5%)	3 (33.3%)	11 (33.3%)	4 (20%)	0.3424^d^
BMI	25.1 (23.6–30.2)	24.5 (23.5–30.1)	26.1 (23.9–30.6)	28.4 (24.8–39.6)	26.5 (23.9–31.8)	26.0 (21.4–29.4)	0.1174^c^
**Preexisting conditions**							
Hypercholesterolemia	41 (12%)	20 (11.2%)	14 (13.7%)	2 (22.2%)	3 (9.1%)	2 (10%)	0.8018^d^
Nicotine use disorder	109 (31.8%)	63 (35.2%)	26 (25.5%)	3 (33.3%)	11 (33.3%)	6 (30%)	0.5746^d^
Coronary heart disease	37 (10.8%)	20 (11.2%)	9 (8.8%)	1 (11.1%)	5 (15.2%)	2 (10%)	0.8937^d^
Hypertension	124 (36.2%)	61 (34.1%)	37 (36.3%)	3 (33.3%)	16 (48.5%)	7 (35%)	0.6355^d^
Liver cirrhosis or chronic hepatitis	21 (6.1%)	18 (10.1%)	2 (2%)	0 (0%)	1 (3%)	0 (0%)	**0.0352** ^**d**^
Chronic kidney disease	23 (6.7%)	11 (6.1%)	9 (8.8%)	1 (11.1%)	2 (6.1%)	0 (0%)	0.6339^d^
Diabetes mellitus	49 (14.3%)	27 (15.1%)	13 (12.7%)	0 (0%)	6 (18.2%)	3 (15%)	0.6975^d^
Oncological disorders	59 (17.2%)	34 (19%)	18 (17.6%)	0 (0%)	4 (12.1%)	3 (15%)	0.5655^d^
Immunodeficiency	93 (27.1%)	56 (31.3%)	27 (26.5%)	3 (33.3%)	4 (12.1%)	3 (15%)	0.1353^d^
Chronic lung disease	97 (28.3%)	50 (27.9%)	29 (28.4%)	3 (33.3%)	9 (27.3%)	6 (30%)	0.9966^d^
CPR within 48 h before EMCO	32 (9.3%)	23 (12.8%)	7 (6.9%)	0 (0%)	2 (6.1%)	0 (0%)	0.1494^d^
**Respiratory situation before ECMO**							
Horowitz index	70 (58–93)	72 (60–107)	69 (58–87)	70 (61–102)	63 (49–77)	65 (46–96)	**0.0091** ^**c**^
pO_2_-arterial (mmHg)	65 (58–75)	66 (58–78)	64 (56–71)	70 (59–80)	62 (44–71)	61 (45–73)	**0.0164** ^**c**^
FiO_2_	1.0 (0.8–1.0)	1 (0.8–1)	1 (0.8–1)	1 (0.9–1)	1 (1–1)	1 (0.8–1)	0.4644^c^
pCO_2_-arterial (mmHg)	56 (46–73)	56 (46–72)	59 (45–78)	51 (41–115)	54 (47–71)	48 (40–71)	0.6284^c^
pH–arterial	7.3 (7.2–7.3)	7.2 (7.2–7.3)	7.3 (7.2–7.3)	7.3 (7.2–7.4)	7.4 (7.2–7.5)	7.3 (7.3–7.4)	**0.0030** ^**c**^
Peak inspiratory pressure ≥42 cm H_2_O	33 (9.6%)	24 (13.4%)	6 (5.9%)	1 (11.1%)	0 (0%)	2 (10%)	0.0869^d^
**ICU stay**							
Duration of ICU stay from ECMO d1 (d)	13.7 (8.7–26.8)	11.0 (5.9–18.0)	17.0 (9.5–29.7)	26.7 (14.4–57.3)	17.8 (10.8–42.8)	40.8 (21.7–72.6)	**0.0001** ^**c**^
ECMO runtime (d)	7.9 (4.7–15.0)	147 (80–239)	230 (147–413)	615 (169–852)	422 (220–778)	782 (407–1664)	**0.0001** ^**c**^
Mechanical ventilation (d)	11.8 (6.7–23.8)	9.5 (5.3–15.3)	14.8 (8.7–26.4)	26.7 (10.0–38.9)	19.1 (10.5–45.2)	38.1 (5.7–72.6)	**0.0001** ^**c**^
**Endpoints**							
30-day survival	179 (52.2%)	80 (44.7%)	55 (53.9%)	6 (66.7%)	22 (66.7%)	16 (80%)	**0.0077** ^**d**^
ICU survival	160 (46.6%)	78 (43.6%)	49 (48.0%)	4 (44.4%)	17 (51.5%)	12 (60%)	0.6406^d^
Hospital survival	159 (46.4%)	78 (43.6%)	48 (47.1%)	4 (44.4%)	17 (51.5%)	12 (60%)	0.6553^d^
ECMO weaning	197 (57.4%)	103 (57.5%)	56 (54.9%)	5 (55.6%)	19 (57.6%)	14 (70%)	0.8136^d^
Vent. Weaning	169 (49.3%)	80 (44.7%)	51 (50.0%)	5 (55.6%)	19 (57.6%)	14 (70%)	0.1981^d^

Data given in median (interquartile range) or in number of patients patients (percentage of group). p-values are calculated between groups using either ^c^Kruskal–Wallis test or ^d^Chi-Square test.

BMI, body-mass-index; CPR, cardiopulmonary resuscitation; ECMO, extracorporeal membrane oxygenation; Vent., ventilation. Bold values indicate a p-value < 0.05.

The authors apologize for this error and state that this does not change the scientific conclusions of the article in any way. The original article has been updated.

