# Supplementary material for: Survival outcomes and mobilization during venovenous extracorporeal membrane oxygenation: a retrospective cohort study
Source: Front Med (Lausanne). 2023 Sep 28;10:1271540. doi: 10.3389/fmed.2023.1271540 (PMC10569171; doi:10.3389/fmed.2023.1271540)
Supplement: Supplementary file 1 [file Presentation_1.pdf]

# Supplementary material

**Supplementary figure 1: Outcome in V-V ECMO according to mobilization.**

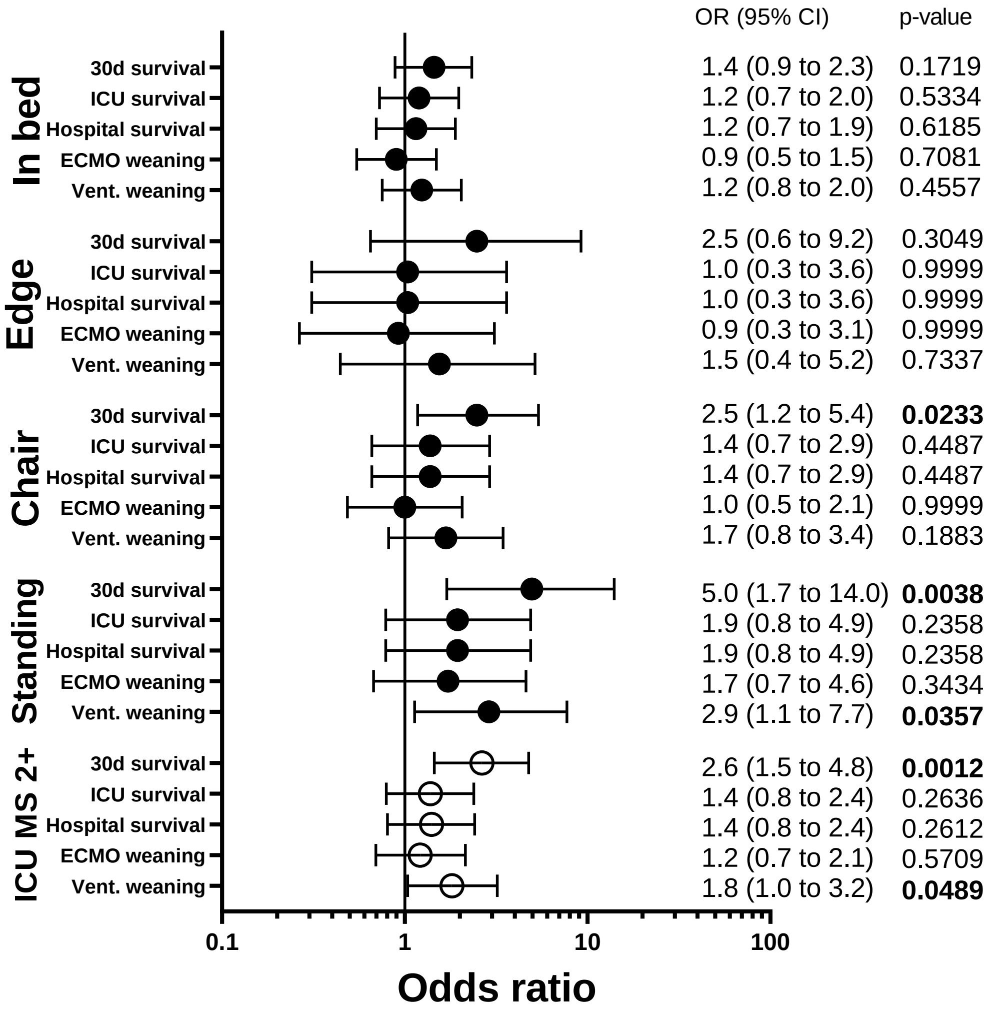


Each mobilization (In bed, Edge (of bed), Chair, Standing) was compared to patients without mobilization (None) or to those with a lower ICU mobility scale (ICU MS 2+) and an OR for each outcome was calculated. A Odds ration >1 indicates better outcome with mobilization. Abbreviations: OR = odds ratio; CI = confidence interval; ICU = intensive care unit; ICU MS 2+ = ICU mobility scale ≥2; Vent. = Ventilation

**Supplementary figure 2: Outcome in V-V ECMO according to mobilization.**

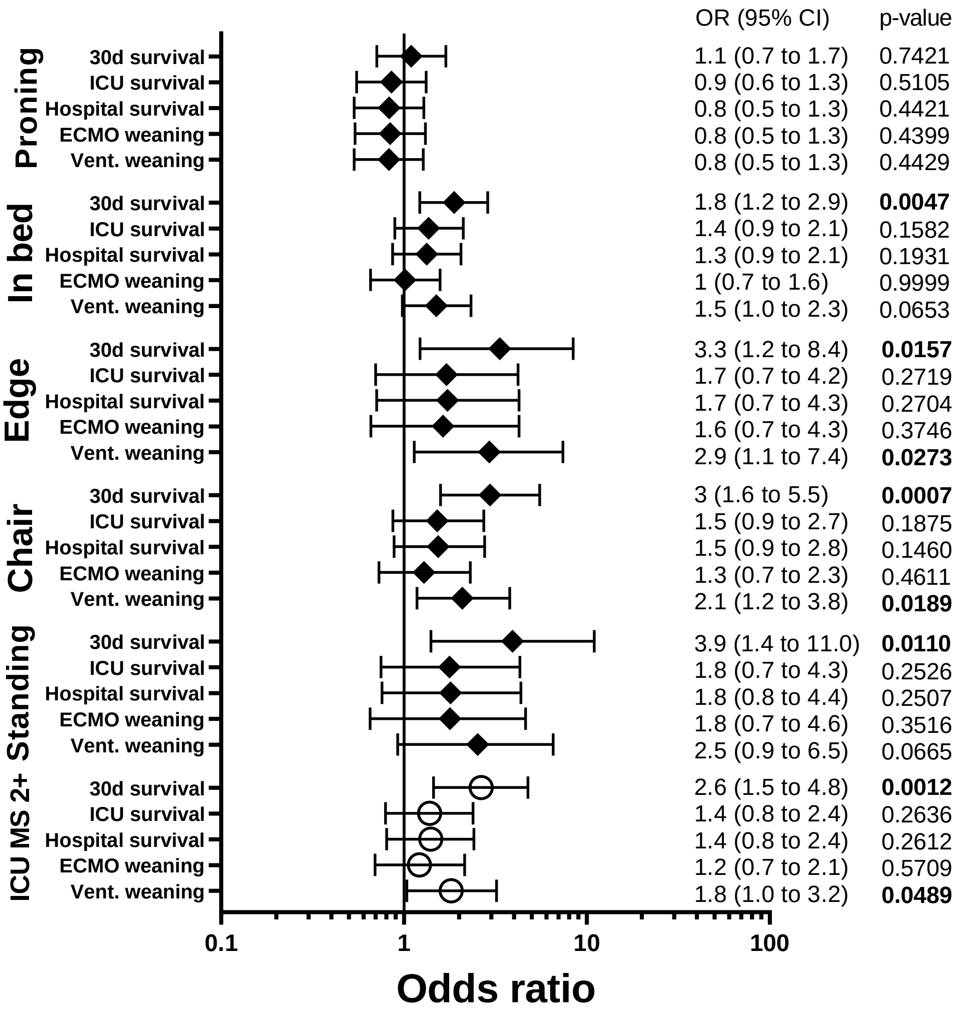


Each mobilization (Proning, In bed, Edge (of bed), Chair, Standing) was compared to all patients not receiving this particular type of mobilization or to those with a lower ICU mobility scale (ICU MS 2+) and an OR for each outcome was calculated. A Odds ration >1 indicates better outcome with mobilization. Abbreviations: OR = odds ratio; CI = confidence interval; ICU = intensive care unit; ICU MS 2+ = ICU mobility scale ≥2; Vent. = Ventilation
